# Identification of another module involved in the horizontal transfer of the *Haemophilus* genomic island ICE*Hin1056*

**DOI:** 10.1016/j.plasmid.2013.05.008

**Published:** 2013-09

**Authors:** Mario Juhas, Ioanna Dimopoulou, Esther Robinson, Abdel Elamin, Rosalind Harding, Derek Hood, Derrick Crook

**Affiliations:** a Clinical Microbiology and Infectious Diseases, NDCLS, University of Oxford, OX3 9DU, UK; b Departments of Zoology and Statistics, University of Oxford, OX1 3TG Oxford, UK; c Molecular Infectious Diseases Group, The Weatherall Institute of Molecular Medicine, University of Oxford, OX3 9DS Oxford, UK

**Keywords:** Horizontal gene transfer, Genomic island, Conjugation, ICE, *Haemophilus*

## Abstract

A significant part of horizontal gene transfer is facilitated by genomic islands. *Haemophilus influenzae* genomic island ICE*Hin1056* is an archetype of a genomic island that accounts for pandemic spread of antibiotics resistance. ICE*Hin1056* has modular structure and harbors modules involved in type IV secretion and integration. Previous studies have shown that ICE*Hin1056* encodes a functional type IV secretion system; however, other modules have not been characterized yet. Here we show that the module on the 5′ extremity of ICE*Hin1056* consists of 15 genes that are well conserved in a number of related genomic islands. Furthermore by disrupting six genes of the investigated module of ICE*Hin1056* by site-specific mutagenesis we demonstrate that in addition to type IV secretion system module, the investigated module is also important for the successful conjugal transfer of ICE*Hin1056* from donor to recipient cells.

## Highlights

• The investigated module on the 5′ extremity of ICE*Hin1056* consists of 15 genes.
• Genes of this module are homologues of DNA replication and stabilization genes.
• This module is well conserved in a number of genomic islands.
• This module is important for the conjugal transfer of ICE*Hin1056*.

## 1 Introduction

Horizontal gene transfer plays an important role in the diversification and adaptation of microorganisms. A significant part of horizontal gene transfer is facilitated by genomic islands (Dobrindt et al., 2004). Genomic islands harbor genes that play a key role in the evolution of a wide variety of pathogenic and environmental bacteria. These include antibiotic resistance and virulence genes involved in generation of “superbugs” (Juhas et al., 2009; Schubert et al., 2009), as well as catabolic gene clusters and genes involved in the formation of new metabolic pathways (Gaillard et al., 2006; Juhas et al., 2009). A number of genomic islands encode type IV secretion systems (T4SS) used for the delivery of effector proteins into host cells and for the conjugative transfer of DNA from donor to recipient cells (Christie et al., 2005; Juhas et al., 2008; Llosa et al., 2009; Ninio et al., 2009; de la Cruz et al., 2010).

Work over past years has provided evidence that horizontal transfer of antibiotic resistant genes in *Haemophilus* is facilitated by the ICE*Hin1056* family of genomic islands (Dimopoulou et al., 1997, 2002; Juhas et al., 2007). Prior to the 1970’s *Haemophilus influenzae* was universally susceptible to ampicillin; however, shortly after, the first ampicillin and multidrug resistant strains emerged and spread around the globe. The *H. influenzae* genomic island ICE*Hin1056* (NCBI reference sequence: NC011409.1, GI: 209947517) is an exemplar of a genomic island responsible for pandemic spread of antibiotic resistance. ICE*Hin1056* has 59.4 kb, harbors 64 putative open reading frames (ORFs) and encodes resistance to several antibiotics, namely ampicillin, tetracycline, and chloramphenicol. ICE*Hin1056* belongs to integrative and conjugative elements (ICEs), a class of self-transmissible mobile genetic elements that encode apparatus for their own excision from the host’s chromosome, conjugation and reintegration into the chromosome (Wozniak and Waldor, 2010; Seth-Smith et al., 2012).

ICEs, including ICE*Hin1056*, have often modular structure with genes responsible for each function (excision and reintegration, horizontal gene transfer) clustered into distinctive modules (Burrus and Waldor, 2004; Boyd et al., 2009). ICE*Hin1056* subfamily of ICEs harbors module responsible for excision and reintegration into the new host’s chromosome (Sentchilo et al., 2003, 2009; Klockgether et al., 2007), in addition to novel conjugation module (Juhas et al., 2007, 2009). Conjugation systems constitute a subfamily of the T4SSs used by bacteria in the process of conjugative transfer of DNA from donor to recipient cells (Christie et al., 2005), thus contributing to evolution of pathogens (Juhas et al., 2008). Previous work has shown that 24 genes of ICE*Hin1056* designated *tfc1–tfc24* encode a novel T4SS that is evolutionarily distant from the archetypal F and P-like (type IVA) T4SSs and I-like (type IVB) T4SSs (Juhas et al., 2007). This novel GI-like T4SS-encoding conjugation module has been also identified in a number of other genomic islands from a broad spectrum of bacteria, including pKLC102 and PAPI of *Pseudomonas aeruginosa* (Klockgether et al., 2007; Wurdemann andTummler, 2007)*,* the *clc* element of *Pseudomonas* sp. B13 (Gaillard et al., 2008), and SPI-7 of *Salmonella enterica* serovar Typhi (Baker et al., 2008). Preliminary bioinformatics analyses have revealed that in addition to excision/integration and T4SS modules, ICE*Hin1056* harbors antibiotic resistance conferring transposons and a number of other genes whose function has been not identified yet (Mohd-Zain et al., 2004; Juhas et al., 2007, 2007, 2009). ICE*Hin1056* conjugation module is crucial for the dissemination of ICE*Hin1056* as mutants did not produce the T4SS pilus and had reduced conjugation frequencies (Juhas et al., 2007). The other modules of ICE*Hin1056* have not been characterized yet.

In this study we analyse the gene components of the module on the 5′ extremity of ICE*Hin1056*. Furthermore, we investigate the importance of these genes for the successful conjugal transfer of ICE*Hin1056* from donor to recipient cells.

## 2 Materials and methods

### 2.1 Bacterial strains, plasmids and growth conditions

All bacterial strains and plasmids used in this study are listed in Table 1. *H. influenzae* was routinely grown on HIB medium (Columbia agar containing 15 μg/ml NAD and 15 μg/ml hemin), supplemented with appropriate antibiotics. Liquid cultures of *H. influenzae* were grown in brain heart infusion broth (BHI) supplemented with 10 μg/ml NAD, 15 μg/ml hemin and appropriate antibiotics. Liquid cultures were incubated at 200 r.p.m. on a rotatory shaker at 37 °C, while the plate cultures were grown for up to 48 h at 37 °C in an atmosphere containing 5% CO_2_. *Escherichia coli* strains were grown in Luria–Bertani broth (LB), supplemented with appropriate antibiotics (Juhas et al., 2007).

### 2.2 PCR amplification and recombinant DNA methodology

DNA modifying enzymes (New England Biolabs), including restriction and ligation enzymes were used according to manufacturer’s recommendations. Oligonucleotide primers were synthesized by Operon, while Taq DNA polymerase and ProofStart DNA polymerase for PCR amplifications were obtained from Qiagen. All recombinant DNA techniques were performed using standard protocols (Sambrook et al., 1989).

### 2.3 Generation of *H. influenzae* mutant strains

Mutants of the *H. influenzae* ICE*Hin1056* were generated by the method described earlier (Juhas et al., 2007). Briefly, the investigated region of ICE*Hin1056* was cloned into pGEM-TEasy vector (Promega) and subsequently, six genes of this region, namely: *orf3, orf4, orf9, orf10, orf11* and *orf12* were disrupted by insertion of the cassette consisting of the DNA uptake sequence (Smith et al., 1995) and kanamycin resistance gene. Recombinant plasmids were confirmed by PCR and digestion with restriction endonucleases. Plasmids with confirmed insertion of the kanamycin resistance cassette in the investigated genes were linearized by restriction endonuclease digestion. Subsequently 3 μg of the appropriate linearized plasmid constructs were used to transform *H. influenzae* strain Rd11 harboring ICE*Hin1056*. Mutants were generated by reciprocal recombination between ICE*Hin1056* and the introduced mutated DNA sequence. Successful transformants were selected on HIB medium with kanamycin.

### 2.4 Conjugal transfer of the genomic island ICEHin1056

The conjugal transfer efficiency of ICE*Hin1056* was determined by the method described earlier (Juhas et al., 2007). Briefly, approximately 10^8^ bacterial cells grown for 48 h on HIB agar were scraped off the plate and re-suspended in 1 ml BHI. 10 μl of donor cells and 100 μl of recipient cells were mixed, transferred to antibiotic free HIB agar plates and incubated for 6 h. Following incubation, bacteria were harvested, serially diluted in fresh BHI broth and plated on agar plates with appropriate selective antibiotics to determine the number of donors, recipients and transconjugants. Transconjugants and recipients were purified on agar plates containing 10 μg/ml kanamycin +2 μg/ml tetracycline and 2 μg/ml tetracycline, respectively. Conjugation frequencies were calculated as number of transconjugants divided by the number of recipients. Experiments were carried out in triplicate and the mean value and standard error for each strain were calculated.

### 2.5 DNA sequence analysis

Visualization and annotation of ORFs of the investigated region of *H. influenzae* island ICE*Hin1056* and other genomic islands was performed with the help of Artemis: Genome Browser and Annotation Tool (Rutherford et al., 2000). Sequence similarity searches using the BLASTN and BLASTX algorithm and position-specific iterated BLAST (PSI-BLAST) algorithms (Altschul et al., 1990, 1997) were performed by interrogating the National Center for Biotechnology Information (NCBI) website. The Artemis comparison tool (ACT) (Rutherford et al., 2000) was used to visually compare modules of different genomic islands and to identify regions of homology using the TBLASTX algorithm.

## 3 Results and discussion

### 3.1 Identification of the module on the 5′ extremity of ICEHin1056

**Fig. 1:**
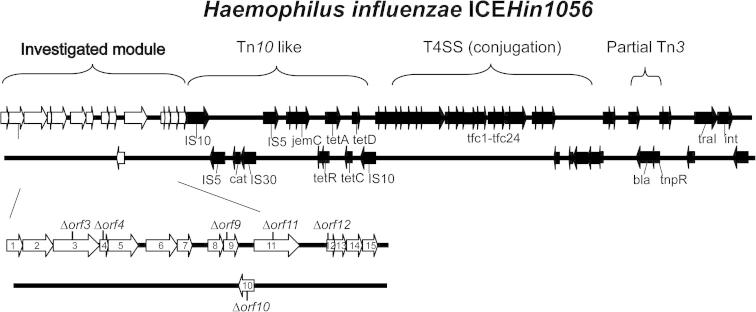
*Haemophilus influenzae* genomic island ICE*Hin1056*. Schematic view of the *H. influenzae* genomic island ICE*Hin1056* showing location of the investigated module (highlighted white). Six genes (*orf3, orf4, orf9, orf10, orf11, orf12*) of the 15 ORFs of the investigated ICE*Hin1056* module were disrupted in this study.

We have shown previously that the conjugation module encoding novel T4SS is important for transfer of ICE*Hin1056* from donor to recipient cells (Juhas et al., 2007); however, other ICE*Hin1056* modules have not been characterized yet. Preliminary bioinformatics analyses suggested that several of the 64 ORFs clustered at the 5′ end of ICE*Hin1056* could play a role in DNA replication and stabilization (Mohd-Zain et al., 2004; Juhas et al., 2007). DNA sequence similarity searches using the BLASTN and TBLASTX algorithms and position-specific iterated BLAST (PSI-BLAST) were performed to identify components of the investigated ICE*Hin1056* module. This *in silico* analysis suggests that the module on the 5′ extremity of ICE*Hin1056* consists of 15 ORFs designated *orf1–orf15* (Fig. 1 and Table 2). These 15 ICE*Hin1056* ORFs are homologous to genes involved in DNA stabilization. *Orf1* is homologous to *parA*, encoding the chromosome partitioning protein important for cell division and plasmid partitioning (Gerdes et al., 2010). *Orf2* is homologous to *dnaB*, which encodes helicase responsible for unwinding of the DNA strands during replication (Itsathitphaisarn et al., 2012). *Orf3* encodes chromosome partitioning protein *parB* involved in segregation of DNA replication products into cells (Gruber and Errington, 2009). *Orf5* and *orf8* are homologous to *ssb*, encoding a single-stranded DNA-binding protein that prevents re-annealing of DNA strands during replication by binding to the lagging strand (Jain et al., 2012). *Orf9* encodes conserved putative lipoprotein, while *orf10* is homologous to *osa* encoding oncogenic suppression system capable of blocking DNA transfer (Lee and Gelvin, 2004). *Orf11* is homologous to *topB* encoding topoisomerase that maintains topological state of DNA during replication (Perez-Cheeks et al., 2012). *Orf12* is homologous to *tonB* providing energy for active transport of substrates (e.g. siderophores) through membrane (Krewulak and Vogel, 2011). *Orf13* is homologous to *traC* encoding DNA primase which plays an important role in DNA replication (Lee et al., 2006). *Orf14* is homologous to *radC* crucial for DNA replication and recombination repair (Attaiech et al., 2008). *Orf15* is homologous to replication protein E1 known to be required for efficient viral DNA replication (Morin et al., 2011). Furthermore, the remaining four genes, namely *orf4, orf6, orf7* and *orf8* also share some degree of homology with chromosome partitioning protein ParA.

These *in silico* findings suggest that the gene cluster under investigation harbors genes involved in ICE*Hin1056* stabilization (Fig. 1).

### 3.2 Investigated ICEHin1056 module is conserved among genomic islands

**Fig. 2:**
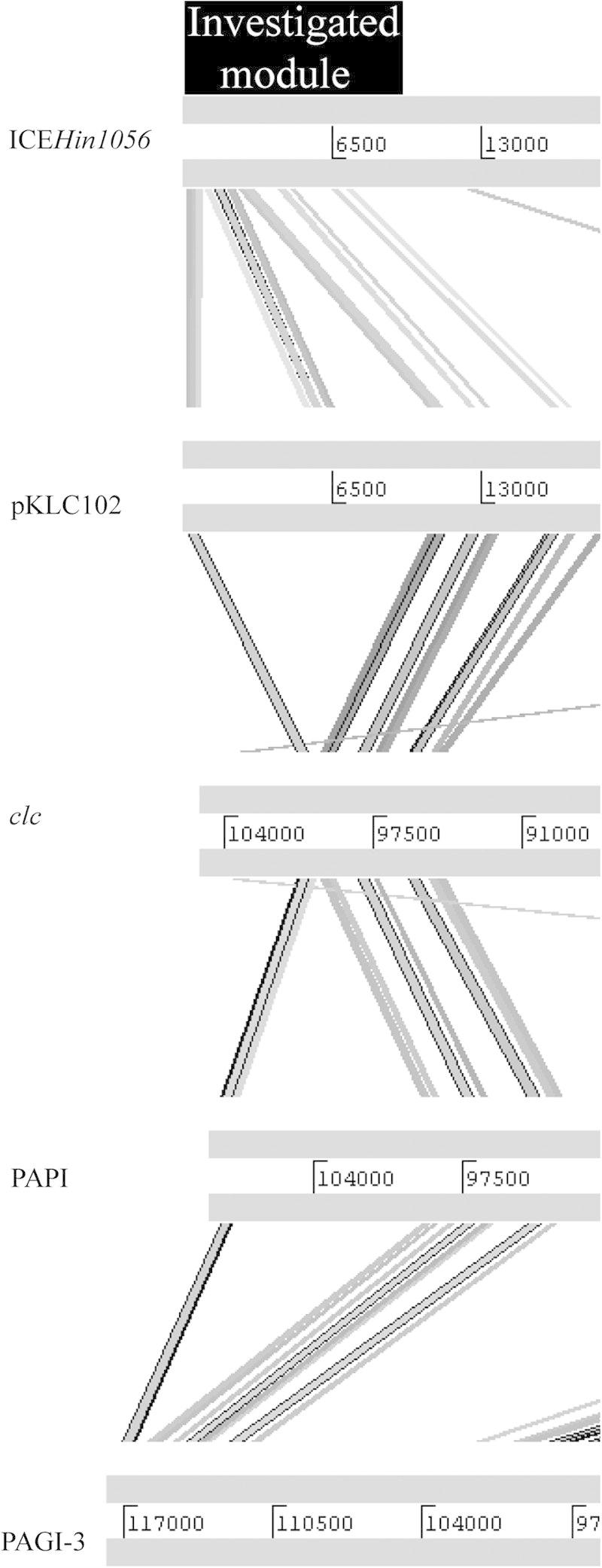
The Artemis Comparison Tool (ACT) analysis of the investigated modules of various genomic islands. Five representative genomic islands involved in dissemination of a wide variety of virulence and metabolic genes have been analysed. The investigated modules of genomic islands tested (highlighted black) share sequence homology. Homologous sequences (minimum cut-off = 50) are indicated by grey lines joining regions of the analyzed genomic islands. The figure shows that the investigated ICE*Hin1056* module is conserved among genomic islands. ORFs which are conserved across genomic islands include *orf1, orf2, orf3, orf4, orf5, orf6, orf7, orf8*, *orf11* and *orf 12.*

ICE*Hin1056* belongs to the diverse family of syntenic genomic islands found in a wide variety of pathogenic and environmental bacteria (Mohd-Zain et al., 2004). Sequence homologies between ICE*Hin1056* and other members of this family of genomic islands revealed that analysed islands share the investigated region, namely genes *orf1, orf2, orf3, orf4, orf5, orf6, orf7, orf8, orf11* and *orf12*. This includes some of the well-known genomic islands responsible for the spread of virulence or catabolic genes among bacteria, namely PAPI, PAGI-3 and pkLC102 of *Pseudomonas aeruginosa*, the *clc* element of *Pseudomonas* sp. (Fig. 2), as well as SPI-7 of *S. enterica* serovar Typhi and genomic islands of *Haemophilus somnus, Haemophilus ducreyi, Pseudomonas fluorescens, Photorhabdus luminescens, Xanthomonas axonopodis, Ralstonia metallidurans* and *Yersinia enterocolitica*. We have identified the investigated module also in a number of other bacteria, including *Erwinia carotovora* atroseptica SCRI1043, *Legionella pneumophila, Azoarcus* sp. EbN1 and *Pseudomonas syringae pv. Phaseolica*. Furthermore, the investigated module was found to be among the most conserved parts of 7 closely related genomic islands of *H. influenzae* and *Haemophilus parainfluenzae* (Juhas et al., 2007), as well as in a number of mobile genetic elements of the SPI-7 family of integrative and conjugative elements within *Enterobacteriaceae* (Seth-Smith et al., 2012). It is plausible to assume that with the increasing number of whole genome sequences available in the public databases, the investigated module will be identified in other genomic islands and bacterial species in the future.

### 3.3 Genes of the investigated ICEHin1056 module are important for conjugation

We have shown previously that disruption of genes of the ICE*Hin1056* T4SS module led to the strong reduction (up to 100,000-fold) of the conjugation transfer frequencies. In the parent strain, ICE*Hin1056* was transferred from the donor to the recipient strain of *H. influenzae* at a frequency of 3 × 10^−2^, while the conjugation frequencies of the mutants of the T4SS module ranged from 6 × 10^−3^ to 1 × 10^−7^ (Juhas et al., 2007).

**Fig. 3:**
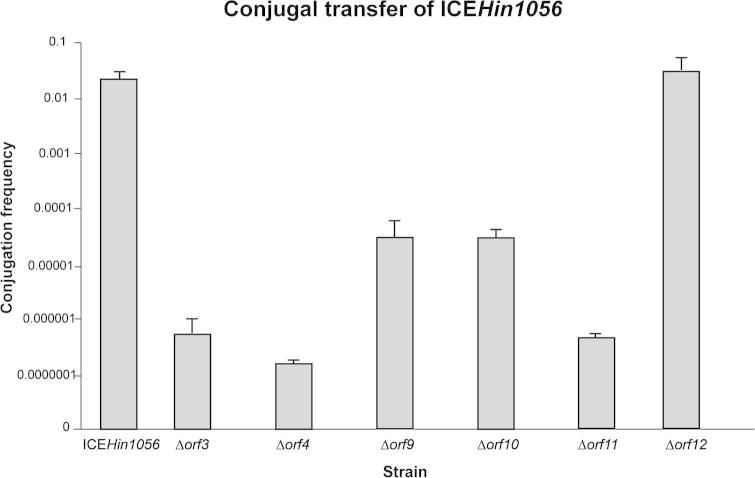
Transfer of the *H. influenzae* genomic island ICE*Hin1056*. Graph showing the conjugation transfer frequencies of the parent strain (ICE*Hin1056*) and mutants in the genes of the investigated module (Δ*orf3*, Δ*orf4*, Δ*orf9*, Δ*orf10*, Δ*orf11*, Δ*orf12*). Mutants transferred less efficiently from donor to recipient and had conjugal transfer frequencies reduced by up to 100,000-fold. The results presented here show the mean values and standard errors calculated from three independent experiments.

We have disrupted six genes of the investigated module on the 5′ extremity of ICE*Hin1056* (*orf3, orf4, orf9, orf10, orf11, orf12*) which had well placed unique restriction sites with the kanamycin resistance cassette (Fig. 1) to investigate the importance of these genes for conjugal transfer of ICE*Hin1056*. Our previous expression analysis has clearly demonstrated that this kanamycin resistance cassette has no polar effect on downstream transcripts and does not interfere with the expression of downstream genes (Juhas et al., 2007). All constructed mutant strains (ICE*Hin1056Δorf3*, ICE*Hin1056Δorf4*, ICE*Hin1056Δorf9*, ICE*Hin1056Δorf10*, ICE*Hin1056Δorf11*, ICE*Hin1056Δorf12*) were tested for their ability to transfer genomic island ICE*Hin1056* by conjugation to the same recipient. As shown in Fig. 3, conjugation frequencies of the analyzed mutants were reduced strongly when compared to the parent strain and ranged from 4 × 10^−2^ to 2 × 10^−7^. Only one mutant, ICE*Hin1056Δorf12*, had conjugal transfer efficiency comparable to the parent strain. ICE*Hin1056Δorf9* and ICE*Hin1056Δorf10* conjugated approximately 1000-times less efficiently than the parent strain, while the remaining three mutants, ICE*Hin1056Δorf3*, ICE*Hin1056Δorf4* and ICE*Hin1056Δorf11*, had conjugal transfer efficiencies reduced strongly by up to 100,000-fold (Fig. 3). Interestingly, the 100,000-fold decrease of the conjugation efficiency of some of the mutants of the investigated region is almost equivalent to the conjugation efficiency of the most affected mutants of the T4SS module, thus confirming that besides T4SS module, these genes are also important for the horizontal transfer of ICE*Hin1056*.

## 4 Conclusions

The investigated module on the 5′ extremity of ICE*Hin1056* consists of 15 ORFs homologous to genes involved in DNA replication and stabilization. The investigated ICE*Hin1056* region is conserved among a wide variety of genomic islands found in a broad spectrum of pathogenic and environmental bacteria. The mutants of the investigated ICE*Hin1056* module are incapable of horizontal transfer from donor to recipient cells. The ability of the majority of the mutants tested to conjugate was impaired and the frequency of transfer was reduced by up to 100,000-fold.

General consensus view held by the majority of researchers until recently was that ICEs and ICE-like elements are incapable of autonomous replication typical for plasmids (Khan, 2005; Pérez-Segura et al., 2013; Rakowski and Filutowicz, 2013) and instead replicate only with the host cell’s chromosome (Burrus and Waldor, 2004). This view has been challenged recently when two ICE-borne genes, namely *nicK* and *helP*, located on the ICE*Bs1* of *Bacillus subtilis* were found to be crucial for replication and stabilization of ICE*Bs1* (Grohmann, 2010; Lee et al., 2010; Thomas et al., 2013). *NicK* and *helP* encoding relaxase and helicase processivity factor, respectively are involved in the autonomous plasmid-like rolling circle replication of ICE*Bs1*, thus contributing to stability of ICE*Bs1* after excision from the chromosome (Grohmann, 2010; Lee et al., 2010; Thomas et al., 2013). Similarly to the investigated ICE*Hin1056* module, *nicK* and *helP* have homologues in many ICEs and often belong to a larger module. Furthermore, besides *nicK* and *helP*, chromosomally-encoded proteins, such as PcrA-type helicases were shown to be required for ICE*Bs1* replication (Thomas et al., 2013). Investigated ICE*Hin1056* module does not harbor *dnaA* and *topA* homologs, thus suggesting that as in the case of ICE*Bs1*, some ICE*Hin1056* replication and stabilization-required genes might be provided by the host’s chromosome.

In conclusion, our findings demonstrate that module located on the 5′ extremity of ICE*Hin1056* is involved in the horizontal transfer and due to conservation may become paradigm also for the other genomic islands.

## 5 Competing interests

The authors declare that they have no competing interests.

## 6 Authors’ contributions

MJ, DH, RH and DC designed the study. MJ, ID, ER and AE sequenced genomic islands and performed sequence analyses. MJ and ID performed functional analyses. MJ, ID, AE, ER, DH, DC and RH evaluated the results. MJ and DC wrote the paper. All authors read and approved the final manuscript.

## Figures and Tables

**Table 1:** Bacterial strains and plasmids used in this work.

Strains and plasmids Strains	Characteristics	Reference
*Haemopilus influenzae*		
Rd	Wild type, laboratory strain	Fleischmann et al. (1995)
ICE*Hin1056* (Rd11)	Rd harboring ICE*Hin1056*	Juhas et al. (2007)
ICE*Hin1056Δorf3*	*orf3* mutant of ICE*Hin1056*	This study
ICE*Hin1056Δorf4*	*orf4* mutant of ICE*Hin1056*	This study
ICE*Hin1056Δorf9*	*orf9* mutant of ICE*Hin1056*	This study
ICE*Hin1056Δorf10*	*orf10* mutant of ICE*Hin1056*	This study
ICE*Hin1056Δorf11*	*orf11* mutant of ICE*Hin1056*	This study
ICE*Hin1056Δorf12*	*orf12* mutant of ICE*Hin1056*	This study

*Escherichia coli*		
DH5α		Lab collection

*Plasmids*		
pGEM-TEasy	Cloning vector	Promega
	F1ori, lacZ, Amp^r^	

**Table 2:** Gene components of the investigated ICE*Hin1056* module.

ICE*Hin1056* ORF	Gene	Size (bp)	Homology
ICE*Hin1056*.01	orf1	836	parA
ICE*Hin1056*.02	orf2	1355	dnaB
ICE*Hin1056*.03	orf3	1688	parB
ICE*Hin1056*.04	orf4	551	parA
ICE*Hin1056*.05	orf5	1223	ssb
ICE*Hin1056*.06	orf6	755	parA
ICE*Hin1056*.07	orf7	488	parA
ICE*Hin1056*.08	orf8	422	ssb
ICE*Hin1056*.09	orf9	521	lipoprotein
ICE*Hin1056*.10	orf10	557	osa
ICE*Hin1056*.11	orf11	2045	topB
ICE*Hin1056*.12	orf12	428	tonB
ICE*Hin1056*.13	orf13	680	traC
ICE*Hin1056*.14	orf14	470	radC
ICE*Hin1056*.15	orf15	479	E1 helicase
